# The effects of obesity and mobility disability in access to breast and cervical cancer screening in France: results from the National Health and Disability Survey

**DOI:** 10.1186/1472-6963-14-S2-P13

**Published:** 2014-07-07

**Authors:** Clémence Bussière, Jonathan Sicsic, Nathalie Pelletier-Fleury

**Affiliations:** 1 CERMES3, INSERM U988, Villejuif, France

## Objectives

We aimed to disentangle the effects of obesity and mobility limitation on cervical and breast cancer screening among community dwelling women.

## Methods

The data source was the French national Health and Disability Survey - Household Section, 2008. The Body Mass Index (BMI) was used to categorize obesity status. We constructed a continuous score of mobility limitations to assess the severity of disability (Cronbach’s alpha=0.84). Logistic regressions were performed to examine the association between obesity, mobility limitations and the use of Pap test (n=8 133) and the use of mammography (n=7 561). Adjusted odds ratios were calculated (AOR).

Interaction terms between obesity and the disability score were included in models testing for effect modifications.

## Results

Compared with non-obese women, the odds of having a Pap test in the past 3 years was 24% lower in obese women (AOR=0.76; 95% CI: 0.65 to 0.89), the odds of having a mammogram in the past 2 years was 23% lower (AOR=0.77; 95% CI: 0.66 to 0.91). Each time the disability score was 5 points higher, the odds of having a Pap test decreases by 20% (AOR=0.96; 95% CI: 0.94 to 0.98), the odds of having a mammogram decreases by 25% (AOR=0.95; 95% CI: 0.94 to 0.97). There was no significant interaction between obesity and disability score.

## Conclusion

Obesity and mobility limitation are independently associated with a lower likelihood of cervical and breast cancer screening. Protective outreach and follow-up are necessary to reduce inequalities and thus to reduce health disparities in these vulnerable and high-risk populations of obese women with disabilities.

